# Assessment of blood changes post-challenge with *Corynebacterium pseudotuberculosis* and its exotoxin (phospholipase D): A comprehensive study in goat

**DOI:** 10.14202/vetworld.2015.1105-1117

**Published:** 2015-09-22

**Authors:** Z. K. H. Mahmood, F. F. Jesse, A. A. Saharee, S. Jasni, R. Yusoff, H. Wahid

**Affiliations:** 1 Department of Veterinary Clinical Studies, Faculty of Veterinary Medicine, Universiti Putra Malaysia, Serdang, Selangor, Malaysia; 2 Department of Microbiology and Pathology, Faculty of Veterinary Medicine, Universiti Malaysia Kelantan, Malaysia

**Keywords:** blood biochemistry, blood changes, caseous lymphadenitis, goat, *Corynebacterium pseudotuberculosis*, hemogram, leukogram, phospholipase D

## Abstract

**Aim:**

There is very little information regarding blood changes during the challenge of phospholipase D (PLD) in goats. Therefore, this experiment was conducted to study the changes in blood after the challenge with *Corynebacterium pseudotuberculosis* and its exotoxin, PLD to fill in the gap of caseous lymphadenitis (CLA) research.

**Materials and Methods:**

Twenty-six crossbred Boer goats aged 12-14 months were divided into 3 groups; the first group n=6 was inoculated with 1 ml phosphate buffered solution s.c. as the control. The second group n=10 was inoculated with *C. pseudotuberculosis* 1 × 10^9^ cfu s.c. The third group n=10 was intravenous injected with PLD 1 ml/20 kg body weight. Serial blood collections were done at 1 h, 3 h, 5 h, 8 h, and 12 h then every 24 h post-inoculation for the first 30 days of the experiment. Subsequently, the blood collection continued twice a week till the end of the experiment (90 days post-challenge).

**Results:**

Both *C. pseudotuberculosis* and PLD treated groups showed significant changes (p<0.05) in red blood cell count, hemoglobin (Hb), packed cell volume, mean corpuscular volume, mean corpuscular Hb concentration, white blood cell count, neutrophils, lymphocytes, monocytes, eosinophils, basophils, globulin, and total plasma proteins. Similarly, both treated groups showed significant changes (p<0.05) in alanine transaminase, alkaline phosphatase, aspartate transaminase, total bilirubin, calcium concentration, creatine phosphokinase, creatinine, gamma-glutamyl transpeptidase, urea concentration, lactate dehydrogenase, prothrombin time, and activated partial thromboplastin time.

**Conclusion:**

It concluded that *C. pseudotuberculosis* and PLD have a negative impact on the goat’s health in general reflected by all those changes recorded in the hemogram, leukogram, and the blood chemistry.

## Introduction

The blood changes caused by infectious diseases are commonly first discovered during routine blood work. Hemogram, leukogram, and blood biochemistry results give an overall assessment of any pathologically related blood changes. Most of these changes are indirectly caused by the pathogen or by the host response to that pathogen. Nonetheless, the host defense response varies according to many types of the infective bacteria, course of the disease and the type of the inflammatory reaction. Acute bacterial infections commonly lead to neutrophilia whilst chronic bacterial infection may lead to lymphocytosis and monocytosis [1].

Too often, caseous lymphadenitis (CLA) in small ruminants is a subclinical disease that has no clear clinical signs until the lesions become visible in the superficial lymph nodes and are discovered on clinical inspection, yet the infected animals are apparently healthy. Indeed, animals with no obvious CLA lesions do not dismiss the fact that internal abscesses could have developed especially if there is CLA infection in a flock or herd; such deep lesions are only discovered at necropsy [2,3].

CLA decreases production and productivity via lowering the milk and meat production and reduces the value of the hide, and in severe cases fatality may arise of the infected animal itself resulting in financial loss and even rendering the sheep and goats rearing unprofitable [4,5]. Extraordinarily, CLA has a long incubation period ranging between 3 and 20 weeks. However, shorter incubation periods have been reported [6-8] during which only a few animals may develop distinct clinical signs such as fever with some other changes in their vitals such as heart and respiratory rates, inappetence and decreased food consumption and alteration in the general health condition. Oddly, CLA has no significant changes on hemogram in goats challenged with *C. pseudotuberculosis*, but toll reflected significantly on the leukogram between challenged groups at different sampling time [2,5]. Sheep experimentally challenged with *C. pseudotuberculosis* showed changes in the plasma proteins and the hemogram [9].

Phospholipase D (PLD) hydrolyzes the sphingomyelin in mammalian cell membranes increasing the vascular permeability especially the endothelial layer leading to plasma proteins leakage from the blood into the surrounding tissue space and from there into the lymphatic system. This contributes to the dissemination of *C. pseudotuberculosis* from the primary infection site to other parts of the animal’s body [3].

The blood changes during the course of *C. pseudotuberculosis* infection and PLD treatment were reported in mice model of CLA [10]. However, there is scanty information regarding blood changes during the PLD challenge in goats. Therefore, this study was designed to further understand the blood changes during infection with *C. pseudotuberculosis* and the challenge with PLD to fill the gap of CLA research in goats.

## Materials and Methods

### Ethical approval

The experiment was performed according to the guidelines of the care and use of experimental animals provided by institutional animal care and use committee (IACUC). The experimental procedures were approved by Universiti Putra Malaysia, animal care committee (UPM/FPV/PS/3.2.1.551/AUP-R119).

### Isolation and identification of *C. pseudotuberculosis*

Bacteria were isolated from clinical cases of CLA in goats. Isolates were sent to the Veterinary Laboratory Service Unit, Department of Veterinary Pathology and Microbiology, Faculty of Veterinary Medicine, Universiti Putra Malaysia for identification and confirmation of the bacteria according to principles and methods described in the microbiological diagnostic laboratory (University of California, Davis, Revised Edition 2008).

### PLD extraction

*C. pseudotuberculosis* exotoxin was extracted following the method described by Zaki [11]. Briefly, 2 or 3 loops of a 48 h culture of *C. pseudotuberculosis* were inoculated in a flask of freshly prepared bovine heart-liver medium. The flask was incubated anaerobically for 7 days at 37°C in slanting position of 15-20°. The culture that developed a pellicle was used. PLD separation started with centrifugation of the culture medium at 8000 rpm/15 min in a refrigerated centrifuge. The supernatant was collected and passed via sterile cellulose membrane filter (0.2 µm) and stored at 4°C then used in the experiment.

### Experimental inoculations

Twenty-six crossbred Boer goats (13 bucks and 13 does) aged between 12 and 14 months with no history of vaccination against CLA were screened twice (3 months apart) for CLA using agar gel immunodiffusion test prior to the experiment.

The goats were divided randomly into 3 groups; the 1^st^ group consisted of 6 goats (3 males and 3 females) housed separately and inoculated with 1 ml phosphate buffered solution subcutaneously as a control. The 2^nd^ group consisted of 10 goats (5 males and 5 females housed separately) was inoculated with *C. pseudotuberculosis* 1 × 10^9^ cfu subcutaneously; the 3^rd^ group also consisted of 10 goats (5 males and 5 females housed separately) injected with PLD 1 ml/20 kg body weight intravenously. Serial blood collections were done at 1 h, 3 h, 5 h, 8 h, and 12 h then every 24 h post-inoculation for the first 30 days of the experiment. Subsequently, the blood collection continued twice a week till the end of the experiment (12 weeks post-inoculation).

### Complete blood count (CBC) and blood biochemistry

The blood samples were collected from the treated groups and the control animals for hematological and blood biochemical analysis. The blood samples were analyzed using Animal Blood Counter special for veterinary use (*abc^™^*), and the serum samples were analyzed using 902 Automatic Analyzer (Hitachi^®^, Japan). The examined parameters were red blood cell count (RBC), hemoglobin (Hb), packed cell volume (PCV), mean corpuscular volume (MCV), mean corpuscular Hb concentration (MCHC), white blood cell count (WBC), neutrophils, lymphocytes, monocytes, eosinophils, basophils, globulin, and total plasma proteins.

Blood biochemistry parameters were alanine transaminase (ALT), alkaline phosphatase (ALP), aspartate transaminase (AST), total bilirubin, calcium concentration, creatine phosphokinase (CPK), creatinine, gamma-glutamyl transpeptidase (GGT), urea concentration, lactate dehydrogenase (LDH), prothrombin time (PT), and activated partial thromboplastin time (APTT).

### Statistical analysis

Statistical analysis was performed using SPSS version 19.0. Repeated measure Analysis of Variance was used to analyze the hemogram, leukogram, and blood biochemistry. All values were reported as mean ± standard error at 95% confidence level.

## Results

CBC is the most common performed blood tests for clinical or research purposes that provide an overview of patient’s general health status and can also indicate the presence of any kind of disease. In any research, monitoring blood biochemical parameters is crucial to detect any changes in an early period and it can be of greater help to anticipate preliminary results, even though there are no obvious symptoms that manifest on clinical inspection. Blood analysis was conducted throughout the experimental period (12 weeks) to ensure complete understanding of CLA pathogenesis via blood profiling.

### RBC count

*C. pseudotuberculosis* infected group showed a significant decrease (p<0.05) in RBC count toward the end of the experiment, distinctively in week 10, week 11, and week 12 compared to the control. PLD challenged group showed no changes in their RBC count (Table-1).

**Table-1:** RBCs count of the goats post-inoculation with *C. pseudotuberculosis* and PLD (mean±SE).

Groups	Control	*C. pseudotuberculosis*	PLD

Weeks	×10^12^/L	×10^12^/L	×10^12^/L
1	13.40±0.47	13.46±0.22	13.10±0.15
2	12.53±0.26	13.24±0.19	12.66±0.19
3	12.20±0.32	13.08±0.13	12.26±0.38
4	11.86±0.26	12.60±0.15	12.06±0.50
5	12.66±0.69	13.10±0.50	11.88±0.70
6	11.76±0.20	12.58±0.64	11.71±0.95
7	11.70±0.17	12.28±1.11	11.82±0.99
8	12.03±0.37	12.60±1.00	12.34±0.92
9	12.76±0.33	12.14±1.04	11.91±0.65
10	12.10±0.36	9.92±2.48*	12.28±0.49
11	12.36±0.33	10.06±2.53*	12.30±0.38
12	11.93±0.54	9.98±2.50*	12.00±0.40

* Significant value p<0.05. Comparison between inoculated groups and the control, *C. pseudotuberculosis=Corynebacterium pseudotuberculosis*, SE=Standard error, PLD=Phospholipase D

### Hb concentration

Hb concentration was significantly decreased (p<0.05), in *C. pseudotuberculosis* infected group in week 10, week 11, and week 12 compared to PLD challenged group and to the control (Table-2).

**Table-2:** Hb concentration of the goats post-inoculation with *C. pseudotuberculosis* and PLD (mean±SE).

Groups	Control	*C. pseudotuberculosis*	PLD

Weeks	g/L	g/L	g/L
1	94.13±2.79	102.22±3.94	99.20±1.70
2	93.00±4.56	103.36±3.53	93.70±2.70
3	88.93±3.75	97.48±2.68	91.48±5.57
4	85.30±3.66	90.94±3.15	88.16±5.58
5	80.70±3.62	92.42±5.78	87.62±7.03
6	79.30±3.34	87.86±7.11	82.30±7.48
7	78.50±3.97	85.12±9.20	81.44±7.17
8	80.03±1.24	89.44±8.89	89.36±7.57
9	88.06±2.61	86.06±8.02	85.12±5.08
10	80.40±4.40	65.32±16.33*	85.72±4.18
11	82.90±3.79	69.36±17.42*	86.64±3.40
12	80.86±6.38	69.16±17.63*	83.32±3.94

* Significant value p<0.05. Comparison between inoculated groups and the control, *C. pseudotuberculosis=Corynebacterium pseudotuberculosis*, SE=Standard error, PLD=Phospholipase D, Hb=Hemoglobin

### PCV

*C. pseudotuberculosis* infected group showed a significant (p<0.05) increase in packed cell volume in week 2, week 3, and week 5 compared to the control, whilst PLD challenged group showed a significant (p<0.05) increase in week 10 compared to the control (Table-3).

**Table-3:** PCV of the goats post-inoculation with *C. pseudotuberculosis* and PLD (mean±SE).

Groups	Control	*C. pseudotuberculosis*	PLD

Weeks	L/L	L/L	L/L
1	0.24±0.00	0.26±0.01	0.26±0.01
2	0.22±0.01	0.27±0.00*	0.24±0.00
3	0.19±0.01	0.24±0.00*	0.22±0.01
4	0.21±0.00	0.22±0.00	0.19±0.01
5	0.20±0.00	0.27±0.01*	0.21±0.02
6	0.21±0.01	0.22±0.02	0.24±0.00
7	0.21±0.01	0.22±0.02	0.22±0.01
8	0.20±0.00	0.23±0.02	0.22±0.02
9	0.24±0.00	0.24±0.02	0.24±0.01
10	0.19±0.01	0.23±0.02	0.25±0.01*
11	0.22±0.00	0.18±0.04	0.22±0.01
12	0.21±0.01	0.19±0.04	0.22±0.01

* Significant value p<0.05. Comparison between inoculated groups and the control, *C. pseudotuberculosis=Corynebacterium pseudotuberculosis*, SE=Standard error, PLD=Phospholipase D, PCV=Packed cell volume

### MCV

Infection with *C. pseudotuberculosis* showed a significant increase (p<0.05) in week 8 and a significant decrease (p<0.05) in week 11, and week 12 in MCV compared to the control. In PLD challenged group, animals showed a significant increase (p<0.05) in week 8 and week 10 in MCV compared to the control (Table-4).

**Table-4:** MCV of the goats post-inoculation with *C. pseudotuberculosis* and PLD (mean±SE).

Groups	Control	*C. pseudotuberculosis*	PLD

Weeks	fL	fL	fL
1	18.89±0.10	19.87±0.52	19.84±0.80
2	17.51±0.64	20.67±0.48	19.25±0.40
3	17.74±0.26	19.55±0.55	17.88±0.82
4	16.54±1.09	19.50±0.45	18.85±0.46
5	21.50±0.70	19.51±0.46	21.20±0.80
6	17.83±0.61	18.11±1.65	19.85±0.69
7	17.92±1.10	18.76±0.49	18.99±0.47
8	17.19±0.26	20.47±0.60*	20.64±0.48*
9	18.80±0.17	18.22±0.16	17.70±0.48
10	15.68±0.37	14.51±3.63	18.33±0.52*
11	18.05±0.45	14.93±3.74*	18.14±0.60
12	17.53±0.64	13.77±3.45*	16.78±0.51

* Significant value p<0.05. Comparison between inoculated groups and the control, MCV=Mean corpuscular volume, C. pseudotuberculosis=Corynebacterium pseudotuberculosis, SE=Standard error, PLD=Phospholipase D

### MCHC

The MCHC showed a significant decrease (p<0.05) in week 4, week 10, week 11, and week 12 in *C. pseudotuberculosis* infected group compared to the control, and a significant decrease (p<0.05) in week 5 in PLD challenged animals compared to the control (Table-5).

**Table-5:** MCHC of the goats post-inoculation with *C. pseudotuberculosis* and PLD (mean±SE).

Groups	Control	*C. pseudotuberculosis*	PLD

Weeks	g/L	g/L	g/L
1	392.22±6.73	381.81±6.98	383.36±10.88
2	424.24±13.08	377.37±3.56	384.38±5.15
3	410.46±3.93	381.23±3.32	416.77±4.24
4	436.73±18.73	369.67±4.35*	386.59±7.74
5	403.22±8.55	360.41±7.54	346.23±5.29*
6	377.87±5.56	393.88±30.91	353.98±10.54
7	374.96±8.10	363.55±4.27	367.04±8.10
8	387.28±2.74	343.69±3.06	349.83±5.27
9	366.85±2.38	387.72±4.79	404.09±12.39
10	423.09±4.05	290.42±72.70*	381.15±8.42
11	371.04±3.52	292.18±73.38*	381.16±7.81
12	385.73±10.97	294.14±73.77*	373.49±761

* Significant value p<0.05. Comparison between inoculated groups and the control, MCHC=Mean corpuscular hemoglobin concentration, *C. pseudotuberculosis=Corynebacterium pseudotuberculosis*, SE=Standard error, PLD=Phospholipase D

### WBCs count

The group infected with *C. pseudotuberculosis* showed a significant increase (p<0.05) in WBC, 2 times higher than the normal level in week 2 only, and it’s maintained significantly higher (p<0.05) than the control in week 4, week 5, week 8, and week 10. PLD challenged animals showed a significant increase (p<0.05) in WBC in week 8, week 10, week 11, and week 12 compared to the control (Table-6).

**Table-6:** WBC count of the goats post-inoculation with *C. pseudotuberculosis* and phospholipase D (mean±SE).

Groups	Control	*C. pseudotuberculosis*	PLD

Weeks	×10^9^/L	×10^9^/L	×10^9^/L
1	10.49±2.47	11.37±1.87	10.08±1.41
2	11.36±0.38	22.38±1.67*	12.34±1.84
3	12.76±1.78	14.48±1.19	12.00±2.19
4	10.35±0.87	14.28±0.79*	11.28±1.49
5	8.45±0.45	11.48±0.86*	10.10±1.36
6	13.08±2.03	12.38±1.17	11.08±1.49
7	10.86±0.46	12.42±0.75	12.55±2.18
8	9.43±1.77	11.63±1.49*	11.64±2.79*
9	9.41±1.10	9.20±0.79	8.37±1.33
10	5.89±0.72	8.86±2.32*	11.12±2.37*
11	7.81±1.21	9.22±2.35	11.04±1.64*
12	9.49±2.35	8.92±2.23	12.33±3.03*

* Significant value p<0.05. Comparison between inoculated groups and the control, WBC=White blood cell, PLD=Phospholipase D, *C. pseudotuberculosis=Corynebacterium pseudotuberculosis*, SE=Standard error

### Neutrophil count

Infection with *C. pseudotuberculosis* showed a significant increase (p<0.05) in week 2, week 3, week 4, week 5, and week 8 compared to the control, whilst in PLD challenged group, neutrophil count was significantly decreased (p<0.05) in week 1 compared to the control, and significantly increased (p<0.05) toward the end of the experiment, specifically week 10, week 11, and week 12 compared to the control (Table-7).

**Table-7:** Neutrophil count of the goats post-inoculation with *C. pseudotuberculosis* and PLD (mean±SE).

Groups	Control	*C. pseudotuberculosis*	PLD

Weeks	×10^9^/L	×10^9^/L	×10^9^/L
1	6.71±1.79	7.12±1.32	*5.32±0.71
2	5.97±0.09	16.01±1.02*	6.81±1.03
3	7.48±1.44	9.22±1.04*	6.64±1.25
4	5.44±0.48	8.90±0.92*	5.95±0.52
5	4.70±0.26	6.47±0.70*	5.02±0.79
6	7.87±1.52	7.72±1.01	5.48±1.03
7	6.84±0.32	7.31±0.56	6.35±1.63
8	5.27±0.88	7.20±1.28*	6.27±2.34
9	5.97±0.79	5.46±0.37	4.35±1.08
10	3.34±0.39	4.89±1.33	6.02±1.41*
11	4.10±0.79	5.01±1.26	7.84±2.26*
12	3.95±0.28	4.95±1.33	6.74±0.71*

* Significant value p<0.05. Comparison between inoculated groups and the control, PLD=Phospholipase D, *C. pseudotuberculosis=Corynebacterium pseudotuberculosis*, SE=Standard error

### Lymphocyte count

Both, *C. pseudotuberculosis* and PLD challenged animals showed a significant decrease (p<0.05) in week 6 compared to the control. There was significant increase (p<0.05) in week 1, week 5, week 7, and week 10 in *C. pseudotuberculosis* infected group as well as significant increase (p<0.05) in PLD challenged group in week 1, week 5, week 7, week 9, week 10, week 11, and week 12 compared to the control (Table-8).

**Table-8:** Lymphocy count of the goats post-inoculation with *C. pseudotuberculosis* and PLD (mean±SE).

Groups	Control	*C. pseudotuberculosis*	PLD

Weeks	×10^9^/L	×10^9^/L	×10^9^/L
1	2.22±0.37	2.68±0.23*	2.97±0.47*
2	3.37±0.12	3.81±0.37	3.70±0.67
3	3.74±0.61	3.67±0.41	3.70±0.60
4	3.63±0.40	3.70±0.38	3.80±0.75
5	2.66±0.14	3.55±0.36*	3.69±0.85*
6	3.91±0.24	3.19±0.39*	3.07±0.46*
7	2.97±0.22	3.86±0.46*	3.61±0.45*
8	3.21±0.56	3.21±0.32	2.77±0.44
9	2.38±0.38	2.65±0.20	3.72±0.71*
10	1.93±0.21	3.05±0.78*	4.64±0.61*
11	2.62±0.22	2.85±0.72	3.19±0.56*
12	2.59±0.23	3.03±0.77	3.78±0.57*

* Significant value p<0.05. Comparison between inoculated groups and the control, PLD=Phospholipase D, *C. pseudotuberculosis=Corynebacterium pseudotuberculosis*, SE=Standard error

### Monocyte count

*C. pseudotuberculosis* infection showed a significant increase (p<0.05) in monocyte count in week 1, week 2, week 4, week 5, and week 10 compared to the control, whereas challenging with PLD showed a significant increase (p<0.05) in week 4, week 5, week 10, and week 12 with a significant decrease (p<0.05) in week 2, week 6, and week 9 compared to the control (Table-9).

**Table-9:** Monocyte count of the goats post-inoculation with *C. pseudotuberculosis* and PLD (mean±SE).

Groups	Control	*C. pseudotuberculosis*	PLD

Weeks	×10^9^/L	×10^9^/L	×10^9^/L
1	0.47±0.07	0.59±0.14*	0.46±0.07
2	0.90±0.13	1.33±0.24*	0.71±0.14*
3	0.71±0.09	0.78±0.08	0.75±0.16
4	0.61±0.02	0.96±0.13*	0.79±0.15*
5	0.56±0.08	0.79±0.20*	0.84±0.18*
6	0.62±0.13	0.72±0.12	0.50±0.10*
7	0.46±0.02	0.57±0.07	0.54±0.14
8	0.49±0.14	0.56±0.08	0.58±0.24
9	0.50±0.07	0.48±0.07	0.39±0.16*
10	0.25±0.05	0.39±0.09*	0.61±0.15*
11	0.60±0.11	0.51±0.13	0.61±0.07
12	0.52±0.07	0.39±0.10	0.64±0.09*

* Significant value p<0.05. Comparison between inoculated groups and the control, PLD=Phospholipase D, *C. pseudotuberculosis=Corynebacterium pseudotuberculosis*, SE=Standard error

### Eosinophil count

Eosinophil count showed a significant increase (p<0.05) in *C. pseudotuberculosis* infected group in week 5 and week 9, with a significant decrease (p<0.05) in week 2 compared to the control. Challenging with PLD showed a significant increase (p<0.05) in week 1, week 2, and week 4, with a significant decrease (p<0.05) in week 9, week 10, week 11, and week 12 compared to the control (Table-10).

**Table-10:** Eosinophil count of the goats post-inoculation with *C. pseudotuberculosis* and PLD (mean±SE).

Groups	Control	*C. pseudotuberculosis*	PLD

Weeks	×10^9^/L	×10^9^/L	×10^9^/L
1	0.41±0.15	0.43±0.08	0.77±0.18*
2	0.39±0.18	0.22±0.01*	0.69±0.21*
3	0.38±0.10	0.36±0.01	0.44±0.21
4	0.31±0.02	0.24±0.07	0.40±0.08*
5	0.20±0.08	0.29±0.11*	0.21±0.04
6	0.20±0.03	0.27±0.06	0.22±0.08
7	0.21±0.00	0.22±0.06	0.20±0.05
8	0.17±0.09	0.21±0.03	0.16±0.03
9	0.23±0.08	0.31±0.16*	0.14±0.03*
10	0.20±0.07	0.23±0.10	0.13±0.04*
11	0.23±0.03	0.18±0.05	0.16±0.07*
12	0.25±0.06	0.21±0.10	0.18±0.07*

* Significant value p<0.05. Comparison between inoculated groups and the control, PLD=Phospholipase D, *C. pseudotuberculosis=Corynebacterium pseudotuberculosis*, SE=Standard error

### Basophil count

Infection with *C. pseudotuberculosis* showed a significant increase (p<0.05) in basophil count in week 4, week 5, week 6, week 8, week 9, week 10, week 11, and week 12 compared to the control. Moreover, PLD challenged group showed a significant increase (p<0.05) in basophil count between week 1 and week 5 and between week 8 and week 12 compared to the control (Table-11).

**Table-11:** Basophil count of the goats post-inoculation with *C. pseudotuberculosis* and PLD (mean±SE).

Groups	Control	*C. pseudotuberculosis*	PLD

Weeks	×10^9^/L	×10^9^/L	×10^9^/L
1	0.09±0.07	0.11±0.01	0.19±0.08*
2	0.10±0.00	0.12±0.08	0.17±0.05*
3	0.12±0.04	0.13±0.03	0.19±0.07*
4	0.09±0.04	0.17±0.03*	0.15±0.03*
5	0.07±0.02	0.15±0.01*	0.13±0.04*
6	0.10±0.05	0.14±0.02*	0.09±0.02
7	0.10±0.00	0.12±0.00	0.12±0.02
8	0.04±0.02	0.13±0.02*	0.11±0.03*
9	0.05±0.02	0.09±0.00*	0.12±0.04*
10	0.07±0.01	0.12±0.02*	0.21±0.05*
11	0.10±0.02	0.17±0.06*	0.14±0.01*
12	0.06±0.01	0.08±0.02*	0.17±0.01*

* Significant value p<0.05. Comparison between inoculated groups and the control, PLD=Phospholipase D, *C. pseudotuberculosis=Corynebacterium pseudotuberculosis*, SE=Standard error

### ALT

*C. pseudotuberculosis* infection showed a significant decrease (p<0.05) in ALT concentration in week, week 3, week 4, week 5, week 10, and week 11 compared to the control, while PLD challenged group showed a significant increase (p<0.05) in ALT concentration in week 6 only, with a significant decrease (p<0.05) in week 1, week 4, week 5, week 7, week 9, and week 11 compared to the control (Table-12).

**Table-12:** ALT concentration of the goats post-inoculation with *C. pseudotuberculosis* and PLD (mean±SE).

Groups	Control	*C. pseudotuberculosis*	PLD

Weeks	U/L	U/L	U/L
1	24.70±2.58	21.88±1.62*	21.44±1.15*
2	22.83±3.03	20.22±1.11	22.24±2.14
3	22.83±1.67	18.44±1.92*	22.78±3.16
4	25.63±0.55	17.94±0.85*	22.94±2.79*
5	19.00±0.00	15.72±2.03*	16.02±1.77*
6	13.43±6.82	12.56±3.82	19.16±3.28*
7	22.33±1.44	19.72±1.98	17.48±1.73*
8	24.13±1.28	22.86±2.46	23.56±2.64
9	25.06±2.23	24.16±1.84	19.44±2.31*
10	23.80±0.89	16.18±4.06*	23.36±3.29
11	22.40±1.65	15.26±4.06*	18.44±2.37*
12	18.23±2.51	15.26±4.27	18.44±3.62

* Significant value p<0.05. Comparison between inoculated groups and the control, ALT=Alanine transaminase, PLD=Phospholipase D, *C. pseudotuberculosis=Corynebacterium pseudotuberculosis*, SE=Standard error

### ALP

Challenging with both *C. pseudotuberculosis* and PLD showed a significant decrease (p<0.05) in ALP concentration 3 times lower than the control in week 1, week 2, week 3, and week 4. However, week 6 showed 5 times lower in *C. pseudotuberculosis* infected group and 2 times lower in PLD challenged group compared to the control. Moreover, ALP showed 3 times lower than the control in week 7, week 8, week 9, and week 12 with 2 times lower in week 11 compared to the control (Table-13).

**Table-13:** ALP concentration of the goats post-inoculation with *C. pseudotuberculosis* and PLD (mean±SE).

Groups	Control	*C. pseudotuberculosis*	PLD

Weeks	U/L	U/L	U/L
1	412.00±325.51	114.60±29.63*	136.20±23.55*
2	260.00±183.55	94.60±24.16*	133.20±27.06*
3	344.00±281.50	84.20±23.97*	111.20±15.91*
4	308.33±242.83	78.80±25.28*	103.20±25.57*
5	41.00±0.00	56.40±20.89	63.20±19.97
6	255.66±214.16	49.60±24.57*	99.20±37.20*
7	234.66±176.85	88.00±30.01*	74.00±20.56*
8	240.33±180.93	88.60±24.94*	96.20±17.24*
9	298.66±239.17	95.60±33.27*	96.40±18.47*
10	65.00±18.23	76.20±29.47	64.60±13.02
11	163.66±129.18	94.60±39.81*	75.80±31.98*
12	194.00±167.06	67.80±26.93*	134.40±91.95*

* Significant value p<0.05. Comparison between inoculated groups and the control, ALP=Alkaline phosphatase, PLD=Phospholipase D, *C. pseudotuberculosis=Corynebacterium pseudotuberculosis*, SE=Standard error

### AST

The concentration of AST increased significantly (p<0.05) in week 1, week 3, week 4, week 5, and week 7, and decreased significantly (p<0.05) in week 11 post-infection with *C. pseudotuberculosis* compared to the control. In PLD challenged group, AST concentration showed a significant increase (p<0.05) in week 1, week 7, and week 10. Strikingly, AST peaked in week 6 compared to the control (Table-14).

**Table-14:** AST concentration of the goats post-inoculation with *C. pseudotuberculosis* and PLD (mean±SE).

Groups	Control	*C. pseudotuberculosis*	PLD

Weeks	U/L	U/L	U/L
1	74.90±5.68	124.80±34.06*	95.22±8.59*
2	95.20±4.38	116.52±30.24	89.26±11.13
3	88.23±8.05	280.42±178.29*	119.38±17.46
4	104.90±3.05	138.30±50.75*	122.52±18.54
5	92.20±0.00	131.44±36.52*	92.66±7.85
6	117.20±11.97	126.54±51.93	297.52±142.95*
7	104.30±5.60	165.58±53.01*	163.26±30.58*
8	167.33±63.78	141.68±39.16	130.58±12.86
9	123.90±14.76	96.34±29.11	92.92±7.52
10	89.66±5.80	96.90±26.00	119.56±11.68*
11	111.66±8.34	78.48±21.58*	115.34±14.78
12	94.60±6.82	78.76±19.81	112.30±7.57

* Significant value *P*<0.05. Comparison between inoculated groups and the control, PLD=Phospholipase D, *C. pseudotuberculosis=Corynebacterium pseudotuberculosis*, SE=Standard error, AST=Aspartate transaminase

### Total bilirubin concentration

Total bilirubin concentration showed a significant increase (p<0.05) in week 2 and week 9, with a significant decrease (p<0.05) in week 8 post-infection with *C. pseudotuberculosis* compared to the control. Challenge with PLD showed an increased bilirubin concentration significantly (p<0.05) in week 2 and week 9, with a significant decrease (p<0.05) in week 1 and week 11 compared to the control (Table-15).

**Table-15:** Total bilirubin concentration of the goats post-inoculation with *C. pseudotuberculosis* and PLD (mean±SE).

Groups	Control	*C. pseudotuberculosis*	PLD

Weeks	µmol/L	µmol/L	µmol/L
1	1.16±0.53	0.72±0.55	0.20±0.06*
2	1.86±0.26	3.08±0.23*	6.28±2.37*
3	2.36±0.08	2.72±0.24	1.92±0.12
4	1.46±0.23	1.66±0.34	1.82±0.15
5	2.00±0.00	1.66±0.19	2.04±0.24
6	1.86±0.28	1.62±0.47	2.10±0.35
7	1.63±0.13	2.02±0.19	1.46±0.35
8	1.90±0.15	1.12±0.17*	1.70±0.64
9	0.63±0.08	1.82±0.21*	1.70±0.24*
10	1.93±0.23	1.58±0.47	1.60±0.22
11	2.06±0.18	1.52±0.50	1.00±0.20*
12	1.70±0.05	1.52±0.51	1.78±0.15

* Significant value p<0.05. Comparison between inoculated groups and the control, PLD=Phospholipase D, *C. pseudotuberculosis=Corynebacterium pseudotuberculosis*, SE=Standard error

### Calcium concentration

Infection with *C. pseudotuberculosis* showed a significant increase (p<0.05) in calcium concentration in week 7, and a significant decrease (p<0.05) in week 6, week 9, week 10, week 11, and week 12 compared to the control. Calcium concentration showed a significant increase (p<0.05) in week 10 post-challenge with PLD compared to the control (Table-16).

**Table-16:** Calcium concentration of the goats post-inoculation with *C. pseudotuberculosis* and PLD (mean±SE).

Groups	Control	*C. pseudotuberculosis*	PLD

Weeks	mmol/L	mmol/L	mmol/L
1	2.10±0.08	2.22±0.13	2.14±0.08
2	2.37±0.03	2.31±0.06	2.19±0.10
3	2.32±0.08	2.22±0.09	2.48±0.06
4	2.13±0.07	2.02±0.07	2.05±0.04
5	1.98±0.00	1.94±0.09	2.10±0.07
6	2.03±0.05	1.38±0.36*	1.94±0.09
7	1.82±0.07	2.14±0.10*	1.79±0.08
8	2.09±0.06	2.00±0.11	2.03±0.13
9	2.18±0.19	1.32±0.55*	2.19±0.06
10	1.95±0.04	1.24±0.51*	2.27±0.05*
11	2.13±0.01	1.68±0.43*	2.00±0.08
12	2.03±0.17	1.39±0.37*	1.84±0.07

* Significant value p<0.05. Comparison between inoculated groups and the control, PLD=Phospholipase D, *C. pseudotuberculosis=Corynebacterium pseudotuberculosis*, SE=Standard error

### Creatinine concentration

Concentration of the creatinine in *C. pseudotuberculosis* infected animals showed a significant increase (p<0.05) in week 2, week 4l and week 7. In week 3, there was a significant decrease (p<0.05), week 5, week 6, week 9, week 10, week 11, and week 12 compared to the control. PLD challenged group showed a significant increase (p<0.05) in creatinine concentration in week 4 and week 7, and a significant decrease (p<0.05) in week 5, week 8, and week 10 compared to the control (Table-17).

**Table-17:** Creatinine concentration of the goats post-inoculation with *C. pseudotuberculosis* and PLD (mean±SE).

Groups	Control	*C. pseudotuberculosis*	PLD

Weeks	µmol/L	µmol/L	µmol/L
1	129.66±23.38	128.80±15.44	121.40±7.52
2	136.66±12.71	163.80±21.11*	133.60±8.35
3	152.00±15.87	122.40±13.77*	141.40±7.59
4	74.66±37.37	128.60±20.23*	123.20±6.40*
5	141.00±0.00	109.80±14.95*	116.80±9.61*
6	109.33±4.84	82.20±24.33*	99.00±22.37
7	84.00±42.01	126.00±18.51*	125.40±11.77*
8	128.33±9.38	115.00±14.01	108.00±9.20*
9	130.66±8.41	112.80±32.91*	144.60±12.38
10	141.33±12.81	81.40±20.89*	104.80±9.02*
11	114.33±9.59	99.40±25.88*	101.00±8.81
12	113.66±8.98	90.40±23.39*	101.60±9.26

* Significant value p<0.05. Comparison between inoculated groups and the control, PLD=Phospholipase D, *C. pseudotuberculosis=Corynebacterium pseudotuberculosis*, SE=Standard error

### CPK concentration

*C. pseudotuberculosis* infection showed a significant increase (p<0.05) in CPK concentration in week one only and a significant decrease (p<0.05) in week 6, week 9 and week 10 compared to the control, whilst PLD challenged group showed a significant increase (p<0.05) in week 1, week 4, and peaked in week 12, and a significant decrease (p<0.05) in week 6, week 7, week 9, and week 10 compared to the control (Table-18).

**Table-18:** Creatinine concentration of the goats post-inoculation with *C. pseudotuberculosis* and PLD (mean±SE).

Groups	Control	*C. pseudotuberculosis*	PLD

Weeks	U/L	U/L	U/L
1	144.00±28.14	273.20±59.30*	216.80±24.66*
2	183.00±57.00	163.80±34.68	218.74±69.75
3	238.00±21.00	200.40±22.16	190.80±29.72
4	139.66±35.06	202.80±56.44	231.00±62.82*
5	127.00±0.00	193.40±29.08	141.80±16.29
6	537.66±199.35	311.40±111.35*	281.20±64.02*
7	328.33±20.66	331.00±53.46	186.80±53.25*
8	356.00±30.13	308.60±43.98	374.00±85.26
9	390.00±43.01	206.20±30.59*	189.60±26.41*
10	288.00±18.47	176.80±45.36*	205.00±35.11*
11	221.00±36.46	155.60±39.67	256.00±37.20
12	245.33±24.30	227.00±74.20	792.60±304.09*

* Significant value p<0.05. Comparison between inoculated groups and the control, PLD=Phospholipase D, *C. pseudotuberculosis=Corynebacterium pseudotuberculosis*, SE=Standard error

### GGT concentration

Infection with *C. pseudotuberculosis* showed 4 times significant increase (p<0.05) in GGT concentration in week 5, and a significant decrease (p<0.05) in week 11 and week 12 compared to the control. Concentration of GGT concentration was significantly increased (p<0.05) during week 6 to week 10, with a significant decrease (p<0.05) in week 1 and week 2 compared to the control (Table-19).

**Table-19:** GGT concentration of the goats post-inoculation with *C. pseudotuberculosis* and PLD (mean±SE).

Groups	Control	*C. pseudotuberculosis*	PLD

Weeks	U/L	U/L	U/L
1	42.33±6.74	46.75±5.35	32.00±2.70*
2	49.00±8.02	47.75±8.81	31.60±4.67*
3	44.00±5.50	49.25±6.51	44.20±4.12
4	52.00±5.56	59.75±10.50	50.40±8.53
5	44.00±0.00	175.00±123.83*	49.40±6.43
6	48.33±8.35	42.00±15.23	91.40±39.53*
7	47.33±6.83	56.00±6.28	83.40±21.74*
8	53.66±8.56	52.50±5.85	81.60±15.71*
9	53.66±9.33	56.75±8.95	67.40±10.26*
10	44.66±2.84	41.50±16.00	66.40±7.13*
11	55.66±13.16	40.00±16.82*	57.60±3.70
12	49.00±8.73	35.00±14.92*	49.00±4.57

* Significant value p<0.05. Comparison between inoculated groups and the control, PLD=Phospholipase D, *C. pseudotuberculosis=Corynebacterium pseudotuberculosis*, SE=Standard error, GGT=Gamma–glutamyl transferase

### Urea concentration

Urea concentration showed a significant increase (p<0.05) post-infection with *C. pseudotuberculosis* in week 2, week 3, and week 7, and a significant decrease (p<0.05) in week 9 and week 10 compared to the control. Challenged with PLD group showed a significant increase (p<0.05) in urea concentration in week 1, week 2, week 3, week 5, week 7, week 8, week 9, and week 10, and a significant decrease (p<0.05) in week 4 compared to the control (Table-20).

**Table-20:** Urea concentration of the goats post-inoculation with *C. pseudotuberculosis* and PLD (mean±SE).

Groups	Control	*C. pseudotuberculosis*	PLD

Weeks	mmol/L	mmol/L	mmol/L
1	4.42±0.06	5.46±0.50	7.70±0.60*
2	5.25±0.56	7.22±0.27*	9.46±1.27*
3	5.20±1.40	7.00±0.48*	8.86±1.24*
4	7.40±0.26	7.28±0.97	5.60±2.98*
5	6.15±0.45	7.16±1.05	9.60±0.00*
6	6.60±0.23	6.64±1.67	6.50±0.51
7	6.40±0.62	8.30±0.72*	8.76±0.97*
8	5.90±0.51	6.50±1.65	9.06±1.34*
9	7.10±0.42	3.84±2.36*	9.26±1.43*
10	8.27±0.36	4.94±2.18*	10.33±1.87*
11	8.40±0.58	7.44±1.92	9.10±1.15
12	8.67±0.55	7.26±1.83	9.40±1.40

* Significant value p<0.05. Comparison between inoculated groups and the control, PLD=Phospholipase D, *C. pseudotuberculosis=Corynebacterium pseudotuberculosis*, SE=Standard error, GGT=Gamma–glutamyl transferase

### Total protein concentration

Infection with *C. pseudotuberculosis* showed a significant increase (p<0.05) in total protein concentration in week 5 and week 7, and a significant decrease (p<0.05) in week 2, week 6, week 9, and week 10 compared to the control. Moreover, challenged with PLD group showed a significant increase (p<0.05) in total protein concentration in week 5, and a significant decrease (p<0.05) in week 2 and week 10 compared to the control (Table-21).

**Table-21:** Total protein concentration of the goats post-inoculation with *C. pseudotuberculosis* and PLD (mean±SE).

Groups	Control	*C. pseudotuberculosis*	PLD

Weeks	g/L	g/L	g/L
1	67.40±3.43	73.46±2.55	71.26±0.53
2	82.83±2.63	69.10±1.86*	50.32±12.61*
3	74.73±1.72	77.80±3.13	73.44±3.03
4	73.00±2.95	77.56±2.96	71.10±2.15
5	59.90±0.00	72.60±3.98*	70.24±1.36*
6	70.53±3.12	58.10±15.17*	70.65±1.97
7	69.23±3.45	80.28±4.07*	68.52±1.97
8	74.30±4.38	63.26±15.83	78.04±3.83
9	80.86±2.21	14.34±14.34*	73.88±1.80
10	73.66±4.98	35.54±14.59*	46.58±11.79*
11	61.63±6.41	59.22±14.90	63.34±3.56
12	58.70±1.30	57.78±14.82	61.66±5.43

* Significant value p<0.05. Comparison between inoculated groups and the control, PLD=Phospholipase D, *C. pseudotuberculosis=Corynebacterium pseudotuberculosis*, SE=Standard error

### Globulin concentration

Globulin concentration showed a significant increase (p<0.05) post-infection with *C. pseudotuberculosis*, in week 1, week 4, week 5, and week 7, and a significant decrease (p<0.05) in week 2, week 9, and week 10 compared to the control. PLD challenged group showed a significant increase (p<0.05) in globulin concentration in week 1 and week 5, and a significant decrease (p<0.05) in week 2 and week 10 compared to the control (Table-22).

**Table-22:** Globulin concentration of the goats post-inoculation with *C. pseudotuberculosis* and PLD (mean±SE).

Groups	Control	*C. pseudotuberculosis*	PLD

Weeks	g/L	g/L	g/L
1	21.83±4.77	35.38±1.30*	32.80±1.70*
2	48.93±2.31	32.72±2.65*	22.96±5.79*
3	42.16±1.60	45.92±1.92	39.60±2.015
4	37.56±2.31	45.98±2.01*	36.78±1.22
5	29.60±0.00	40.02±2.59*	35.62±1.47*
6	35.66±2.23	37.10±9.54	40.74±2.013
7	38.30±2.99	51.82±2.33*	39.80±1.98
8	42.53±3.58	39.98±10.51	46.16±4.27
9	49.56±1.49	10.16±10.16*	43.34±1.12
10	44.66±6.94	19.66±8.82*	24.04±6.25*
11	31.40±5.68	34.98±9.34	37.06±3.61
12	31.53±2.14	34.70±9.68	39.86±3.48

* Significant value p<0.05. Comparison between inoculated groups and the control, PLD=Phospholipase D, *C. pseudotuberculosis=Corynebacterium pseudotuberculosis*, SE=Standard error

### LDH concentration

Infection with *C. pseudotuberculosis* showed a significant increase (p<0.05) in LDH concentration in week 1, week 2, week 3, and week 7, and a significant decrease (p<0.05) in week 4, week 6, week 8, week 10, week 11, and week 12 compared to the control. PLD challenged group, LDH concentration showed a significant increase (p<0.05) in week 1, week 2, week 6, week 7, and week 12, and a significant decrease (p<0.05) in week 8, week 10, and week 11 compared to the control (Table-23).

**Table-23:** LDH concentration of the goats post-inoculation with *C. pseudotuberculosis* and PLD (mean±SE).

Groups	Control	*C. pseudotuberculosis*	PLD

Weeks	U/L	U/L	U/L
1	719.00±99.05	951.40±233.94*	947.400±70.86*
2	679.33±174.34	1001.60±256.00*	1028.60±86.31*
3	854.66±61.20	1045.80±326.84*	887.80±135.05
4	843.00±59.21	705.80±130.65*	806.00±55.17
5	575.33±74.65	611.80±94.79	579.80±56.83
6	863.66±23.21	624.60±181.94*	1062.00±258.57*
7	718.00±159.34	835.00±78.13*	965.20±140.60*
8	1015.33±197.32	575.60±156.97*	904.00±84.89*
9	800.00±81.87	740.20±32.64	730.60±43.09
10	820.66±19.78	550.40±141.05*	673.60±61.12*
11	758.66±112.38	584.20±149.95*	671.60±58.56*
12	770.66±53.24	620.60±157.54*	962.20±158.24*

* Significant value p<0.05. Comparison between inoculated groups and the control, PLD=Phospholipase D, *C. pseudotuberculosis=Corynebacterium pseudotuberculosis*, SE=Standard error, LDH=Lactate dehydrogenase

### PT

PT was significantly increased (p<0.05) post-infection with *C. pseudotuberculosis* in week 2, week 5, week 8, and week 9, and a significant decrease (p<0.05) in week 1 and week 10 compared to the control. Challenge with PLD also showed a significant increase (p<0.05) in PT in week 1 and week 8, and a significant decrease (p<0.05) in week 10 compared with the control (Table-24).

**Table-24:** Prothrombin time of the goats post-inoculation with *C. pseudotuberculosis* and PLD (mean±SE).

Groups	Control	*C. pseudotuberculosis*	PLD

Weeks	s	s	s
1	48.10±17.45	31.56±3.83*	80.36±54.95*
2	25.70±1.70	40.48±4.88*	33.78±4.60
3	26.26±2.09	26.44±1.00	26.50±0.85
4	24.63±1.61	23.62±1.15	22.28±1.60
5	27.20±2.52	38.82±9.10*	25.10±1.03
6	28.86±1.96	23.24±1.67	24.14±0.70
7	22.00±2.35	23.20±1.08	26.02±1.65
8	25.36±1.38	37.46±2.97*	39.70±4.32*
9	31.66±4.49	40.92±4.76*	33.34±2.49
10	49.86±13.44	27.96±7.19*	41.84±5.52*
11	32.70±2.84	30.94±1.82	26.24±6.72
12	22.50±2.51	19.22±4.82	23.64±0.88

* Significant value p<0.05. Comparison between inoculated groups and the control, PLD=Phospholipase D, *C. pseudotuberculosis=Corynebacterium pseudotuberculosis*, SE=Standard error

### APTT

Infection with *C. pseudotuberculosis* showed a significant increase (p<0.05) in APTT in week 2, week 3, week 8, and week 9, and a significant decrease (p<0.05) in week 6 compared to the control, whilst in PLD challenged group, APTT was significantly increased (p<0.05) in week 1, week 8, week 9, and week 10, and a significant decrease (p<0.05) in week 4, week 5, week 6, and week 11 compared to the control (Table-25).

**Table-25:** Activated partial thromboplastin time of the goats post-inoculation with *C. pseudotuberculosis* and PLD (mean±SE).

Groups	Control	*C. pseudotuberculosis*	PLD

Weeks	s	s	s
1	46.40±12.08	49.62±5.84	91.42±52.31*
2	41.93±8.92	76.14±13.57*	48.74±8.31
3	33.00±3.88	46.42±1.72*	35.68±1.43
4	43.36±5.83	35.26±1.38	29.92±1.62*
5	50.20±10.79	53.70±7.60	32.10±1.68*
6	53.26±4.58	37.04±2.87*	33.36±3.32*
7	36.30±7.97	31.18±1.44	34.02±2.80
8	36.73±3.01	55.92±10.67*	48.78±6.18*
9	34.93±5.99	50.84±9.05*	44.04±0.56*
10	43.96±9.73	39.50±10.16	66.26±10.11*
11	55.23±7.67	52.80±6.43	38.60±12.03*
12	28.20±3.17	24.14±6.15	25.36±0.64

* Significant value p<0.05. Comparison between inoculated groups and the control, PLD=Phospholipase D, *C. pseudotuberculosis=Corynebacterium pseudotuberculosis*, SE=Standard error

## Discussion

Laboratory analysis of bodily fluids such as blood or urine via different means, hematology and blood biochemistry is of great value to validate predictive disease diagnosis and/or prognosis; this applies to infectious diseases, cancer and immunopathy. This study reported a wide range of hematological and blood biochemical changes in induced CLA in goats and compared it with PLD challenged goats. Hematological evaluation reached a tipping point in the current study exposing many significant changes post-challenged with *C. pseudotuberculosis* and PLD. Previous studies [5,10,12-14] reported similar findings of our study where there were significant changes in Hb concentration, RBC count, MCV, and MCHC concentrations. These results could be inferred as harmful effects of *C. pseudotuberculosis* exotoxin, PLD, on endothelial cells of the vascular system disrupting the normal physiology of the hemopoietic system. Moreover, it was hypothesized that thrombocytopenia is one of many reasons for anemia or failure of megakaryocytes response in bone marrow [10,13]. A striking difference was revealed in the current study by PLD inoculated animals where the experimental goats showed no significant changes in RBC count and Hb concentrations. This odd outcome may be indicative of an insufficient single-dose of PLD that was inoculated into the animals. Moreover, it may be due to the absence of the pathogen, named, *C. pseudotuberculosis* and lack of its pathological role in the process of the disease to affect or change the studied parameters.

Although, *C. pseudotuberculosis* infection in goats resulted in mastitis and classical CLA cases, but it did not affect RBC count nor PCV [5]. The result from this study disagreed with Junior *et al*. [5] where PCV showed a significant increase in both *C. pseudotuberculosis* and PLD challenged groups. This can be explained as direct effects of the PLD and/or indirect effects of the *C. pseudotuberculosis* causing substantial degeneration of RBCs membrane leading reticuloendothelial system to remove it from the circulation [12,14]. All the findings from this study indicate the different means by which the PLD and the *C. pseudotuberculosis* affecting the body in general and the blood parameters in particular. In addition, other factors such as sex, age, physiological status of the animal, and the individual variations may also impose critical effects on the overall health status of the goats which in turn reflected in the blood work.

Naturally CLA infected sheep showed a significant increase in WBC count due to the increased neutrophil, monocyte and lymphocyte counts [5,12]. Similarly, in this study WBC, neutrophil, monocyte and lymphocyte counts were significantly high post-inoculation with the *C. pseudotuberculosis* and the PLD. These findings are in accord with previous studies findings [10,12,15] who stated that *C. pseudotuberculosis* resulted in significant increase in the parameters under the study in sheep and mice model of CLA. Theoretically, and from a pathophysiological point of view, inoculation of PLD should not lead to any significant changes in the WBC, neutrophil, monocyte and lymphocyte counts. However, we hypothesize, that PLD inoculation resulted in transient immunosuppression which in turns led to an activation of some of the opportunistic pathogens contributing to the significant increase in WBC, neutrophil, monocyte and lymphocyte counts.

In the current study, eosinophil and basophil counts were significantly changed in both challenged groups. A result that disagreed with Ibtisam [12] and Osman *et al*. [10] both studies have reported no significant changes in basophils count during the course of CLA in sheep or in mice. However, histologically, CLA abscessation in the lymph nodes of sheep and goats showed immense infiltration with neutrophil and to a lesser extent with eosinophils; these eosinophils give the push its greenish shade [16]. A recent study in mice suggests that basophil may involve in cellular immunity as a T-cell regulator to mediate the magnitude of the secondary immune response [17]. This study hypothesized that the significant increase in basophil count is due to the cellular immunity response. Hence, that *C. pseudotuberculosis* is a facultative intracellular pathogen and has the ability to live inside the macrophages.

Blood biochemistry assessment orchestrated with the hemogram and the leukogram revealing various significant changes post-infection with *C. pseudotuberculosis* and PLD challenge in the current study. ALT and AST are the most elevated enzymes in liver diseases. Both enzymes could be elevated before any clinical disease is apparent such as in acute hepatic necrosis and their levels may reach as high as 100 times the normal level with peak activity between 7 and 12 days [18]. In this study, liver enzymes such as ATL, ALP, AST, and GGT concentrations were significantly changed in both the PLD and the *C. pseudotuberculosis* inoculated groups. These findings are agreed with those described by some authors [10,12,18-21] who stated that liver damage, bacterial invasion induced liver damage, hepatotoxicity, obstruction of the biliary tree, disease processes that involved hepatocytes integrity and hemolytic diseases can significantly change ATL, ALP, AST, and GGT serum concentrations. We hypothesize that the high fluctuation in liver enzymes can reflect the severity and/or the chronic nature of the disease affecting the liver. Hence, that *C. pseudotuberculosis* inoculation was led to abscess formation in the liver. In addition, the toxic nature of the PLD had the same role in evoking these liver enzymes (ATL, ALP, AST, and GGT) which indicates rather similar pathophysiological mechanism of the *C. pseudotuberculosis*, even though PLD did not lead to any abscess formation in the liver or any other organs.

The bilirubin metabolic defects resulted in jaundice which is in most cases inherited or due to hemolysis. The bilirubin is the end product of Hb breakdown which excreted in the bile. Whilst urea is the end product of protein catabolism process in the body and it is cleared by the kidneys. Measurement of plasma urea concentration reflects the kidney function. Additionally, creatinine is the end product of creatine phosphate breakdown in the muscles. Both, creatinine and urea are cleared from the body by means of the kidneys through the process of glomerular filtration [18]. In our study, total bilirubin, creatinine, urea, and CPK concentrations were significantly changed in both the *C. pseudotuberculosis* and the PLD challenged goats. This result in contrast with Osman *et al*. [10] who stated that there were no significant changes in direct or total bilirubin post-inoculation with *C. pseudotuberculosis* and PLD in mice model of CLA. However, the findings accords with Ibtisam [12] who reported the significant high level of serum creatinine and blood urea concentrations in sheep naturally infected with CLA. This could be attributed to the degenerative effect of *C. pseudotuberculosis* toxin leading to impairment of renal function. The authors suggested that decreased blood flow into the kidneys could increase the serum creatinine and urea concentrations. CPK was significantly high up to 1000 fold post-inoculation of *C. pseudotuberculosis* and PLD in mice. CPK is an indicator for muscle damage, and its elevation could be attributed to the damage imposed by the bacteria and/or its exotoxin on cardiac or skeletal muscles [10,22,23].

Serum LDH is commonly found in the liver, skeletal muscles, heart, and kidneys. Hence, its level pattern changes due to tissue damage and it can be considered a marker of the instability of cellular integrity or cell death caused by pathological condition [24-26]. In this study, LDH was significantly changed post-inoculation with the *C. pseudotuberculosis* and the PLD. It suggested that LDH is a precise indicator that has a positive association with liver diseases, heart problems, uric acid elevation, and hematocrit, and it is proposed to be a marker for cardiac dysfunction [27]. Elevation in LDH serum concentration may raise from tissue damage by the toxic materials or pathological lesions in the liver, lymph nodes, lung, bone marrow, and spleen and it’s hypothesized that LDH source in such cases may be the inflammatory cells [24]. Since, in this study, the pathophysiological mechanism of *C. pseudotuberculosis* and PLD inoculation involved inflammation, tissue damage, high blood urea, high PCV, and toxicity which reflected as high serum LDH concentration. Moreover, estimation of serum LDH level is a useful tool for detection of acute myocardial infarction and if combined with CPK it makes a good diagnostic method for delayed detection [28]. Therefore, we believe that LDH can be used in CLA diagnosis scheme; since, it’s very sensitive biomarker for tissue damage especially in the lung, liver, and lymph nodes, a major organs that affected by CLA in small ruminant.

Cations, particularly, calcium is the most common element in the body, and 99% of it is found in the skeleton. Calcium disorder is presented into two main metabolic forms, hypocalcemia and hypercalcemia. It is within the realm of science that hypocalcemia is due to fall in free calcium, albumin-bound calcium or both whilst hypercalcemia is due to the influx of the calcium from the calcium pool into the extracellular fluids is more than its efflux [18]. In the current study, calcium concentration showed significant changes in both the *C. pseudotuberculosis* and the PLD challenged goats. These findings disagree with those reported by Osman *et al*. [10] who stated there was no significant change in serum calcium concentration post-inoculation with *C. pseudotuberculosis* and PLD in mice. This could be due to the toxin effect on the kidneys resulting in renal failure and leading to reduced glomerular filtration rate and retaining the electrolytes and some other substances in the blood. Calcium binds to albumin making albumin-bound calcium. Such chronic diseases that lead to lower serum albumin ensure low calcium level by default contribution to hypocalcemia [18]. Hence, the albumin concentration in this study was significantly decreased.

Again CLA is a wasting disease that affects the protein level in the body [3]. Total protein concentration in the current study showed significant changes post-inoculation with *C. pseudotuberculosis* and PLD. These findings disagree with those results described by Osman *et al*. [10] who reported that total protein concentration showed no significant changes in mice challenged with *C. pseudotuberculosis* and PLD. However, albumin level was significantly low and parallel to a significant high level of globulin. The globulin was significantly increased 48 h post-inoculation with both *C. pseudotuberculosis* and PLD in mice model of CLA. Similarly, in our study, globulin concentration showed significant changes in *C. pseudotuberculosis* and PLD challenged groups. This increasing level of globulin could be due to immune response to *C. pseudotuberculosis* infection and the production of antibodies [10,12]. Similarly, we believe that PLD inoculation resulted in antibodies production contributing to the increased level of globulin. However, beta globulins, albumin, and total protein were significantly decreased in CLA infected sheep. This is perhaps due to the liver damage that was inflicted by the pathogen and its toxin directly or indirectly causing inappetence which leads to malnutrition of the affected animals. Hence, albumin level reduction could be as a result of albumin catabolism or because of the leakage of the plasma proteins into surrounding tissues since PLD increases the permeability of vasculature particularly capillaries affecting the total protein concentration [3,12,23].

Thrombin has a crucial role in the coagulation process and is mainly produced by the liver and its vitamin K-dependent coagulation factor [29]. PT and APTT are clotting factors that are commonly utilized in laboratory routines to detect any coagulation disorder [30]. Any deficiencies in these two factors predispose to a hemorrhagic risk. Hence, deficiency or prolonged APTT can considered as a predisposing factor for thrombosis more than hemorrhage [31]. In this study, both PT and APTT were significantly changed post-inoculation with *C. pseudotuberculosis* and PLD. APTT used to evaluate the intrinsic and the common coagulation pathways together with blood clotting abnormalities. Short APPT is considered of no clinical value. However, some literature [32] hypothesized that may escalate the chances of thromboembolism supported by bleeding tendency and hypercoagulable state. Meanwhile, extended or increased APPT indicates sepsis, antiphospholipid antibodies and deficiency of coagulation factors [32]. APTT may be a useful and fast indicator to use in CLA diagnosis to confirm suspected cases, especially it takes only a few seconds to obtain the results and need no special kits.

## Conclusion

*C. pseudotuberculosis* and PLD challenges have led to many significant changes in the hemogram, leukogram, and blood biochemistry. Although, PLD has a key role in CLA pathogenesis, yet when inoculated separately it showed different reactions and responses on the parameters under the study. This may provide better understanding of CLA pathogenesis and the important role of PLD in the disease process. Moreover, this study reflected the crucial role of the PLD in CLA pathogenesis in goat and its effects on different body systems and organs represented by all the changes appeared in the hemogram, leukogram, and blood biochemistry.

## Author’s Contributions

FFJ contributed to the design of the field trial. ZKHM ran the experiment and collected the samples. ZKHM and FFJ analyzed the results and drafted the paper. FFJ, AAS, JS, RY and HW have contribuated to the design of the study, writing the manuscript and coordination of the study. All authors have read and approved the manuscript.
